# Migration-Enhanced Epitaxial Growth of InAs/GaAs Short-Period Superlattices for THz Generation

**DOI:** 10.3390/nano14030294

**Published:** 2024-01-31

**Authors:** Ruolin Chen, Xuefei Li, Hao Du, Jianfeng Yan, Chongtao Kong, Guipeng Liu, Guangjun Lu, Xin Zhang, Shuxiang Song, Xinhui Zhang, Linsheng Liu

**Affiliations:** 1 Guangxi Key Laboratory of Brain-Inspired Computing and Intelligent Chips, School of Electronic and Information Engineering, Guangxi Normal University, Guilin 541004, China; 2 Key Laboratory of Integrated Circuits and Microsystems, Education Department of Guangxi Zhuang Autonomous Region, School of Integrated Circuits, Guangxi Normal University, Guilin 541004, China; 3 Key Laboratory of Nanodevices and Applications, Suzhou Institute of Nano-Tech and Nano-Bionics, Chinese Academy of Sciences, Suzhou 215123, China; 4 Sino Nitride Semiconductor Co., Ltd., Dongguan 523000, China; 5 State Key Laboratory of Superlattices and Microstructures, Institute of Semiconductors, Chinese Academy of Sciences, Beijing 100083, China; 6 School of Physical Science and Technology, Lanzhou University, Lanzhou 730000, China

**Keywords:** photoconductive materials, migration-enhanced epitaxy, short-period superlattices

## Abstract

The low-temperature-grown InGaAs (LT-InGaAs) photoconductive antenna has received great attention for the development of highly compact and integrated cheap THz sources. However, the performance of the LT-InGaAs photoconductive antenna is limited by its low resistivity and mobility. The generated radiated power is much weaker compared to the low-temperature-grown GaAs-based photoconductive antennas. This is mainly caused by the low abundance of excess As in LT-InGaAs with the conventional growth mode, which inevitably gives rise to the formation of As precipitate and alloy scattering after annealing. In this paper, the migration-enhanced molecular beam epitaxy technique is developed to grow high-quality (InAs)_m_/(GaAs)_n_ short-period superlattices with a sharp interface instead of InGaAs on InP substrate. The improved electron mobility and resistivity at room temperature (RT) are found to be 843 cm^2^/(V·s) and 1648 ohm/sq, respectively, for the (InAs)_m_/(GaAs)_n_ short-period superlattice. The band-edge photo-excited carrier lifetime is determined to be ~1.2 ps at RT. The calculated photocurrent intensity, obtained by solving the Maxwell wave equation and the coupled drift–diffusion/Poisson equation using the finite element method, is in good agreement with previously reported results. This work may provide a new approach for the material growth towards high-performance THz photoconductive antennas with high radiation power.

## 1. Introduction

Terahertz waves, with frequencies ranging from 0.1 to 10 THz (wavelengths from 30 μm to 3 mm), have received considerable attention for their broad application prospects in the fields of security, medical imaging, nondestructive testing, and wireless communication [1,2,3,4,5,6,7,8,9]. Terahertz time-domain spectroscopy (TDS), as a representative of terahertz technology, shows significant value in revealing a material’s molecular, vibrational, or rotational characteristics, and the compositional and structural information, as well as the optical properties. The high-power, portable, and cost-effective terahertz radiation sources, particularly the photoconductive antennas (PCAs), are the prerequisite for the advanced THz technology development. The low-temperature-grown GaAs (LT-GaAs), with their sub-picosecond carrier lifetime, high mobility, and resistance, have found extensive application in terahertz generation and detection. Practically, the bulky and costly 800 nm Ti:sapphire femtosecond lasers are often necessarily required for THz radiation generation [10].

To advance the development of THz sources towards low-cost, miniaturization, and easy integration, THz radiation generated by irradiating the 1.55 µm fiber femtosecond laser onto the low-temperature-grown InGaAs (LT-InGaAs) photoconductive antennas has gained increasing attention recently. Notably, Takazato et al. conducted experiments where they irradiated a Be-doped LT-InGaAs photoconductive antenna using a 1.56 µm laser, obtaining a THz spectral range exceeding 2.5 THz [11]. Dietz et al. fabricated a PCA using an InGaAs/InAlAs superlattice by mesa etching process, by which a significant boost of terahertz pulse amplitude was achieved, and an expansion of the bandwidth up to 4 THz was obtained [12]. Subsequently, Dietz et al. successfully enhanced the radiation power of an InGaAs/InAlAs PCA to 64 μW through process optimization [13]. However, the significantly lower mobility and resistivity of LT-InGaAs compared to LT-GaAs are not favorable for the further development of the LT-InGaAs PCA. It is well known that the resistivity, mobility, and carrier lifetime of semiconductor materials are largely affected by epitaxial growth conditions such as temperature and excess As content, as well as the annealing process. The migration-enhanced epitaxy (MEE) growth of the group III-V molecular beam epitaxy (MBE) by alternatively depositing group III and group V atoms has been demonstrated to obtain excellent homogeneity and interfacial abruptness [14,15]. In this paper, the MEE technique incorporated with MBE has been developed to grow (InAs)_m_/(GaAs)_n_ short-period superlattices (SLs) instead of InGaAs on InP substrates. The improved mobility and resistivity compared with LT-InGaAs are successfully obtained. The (InAs)_m_/(GaAs)_n_ SLs prepared by the MEE technique in this work provide alternative advantageous PCAs for terahertz technology development.

## 2. Materials and Methods

**Figure 1:**
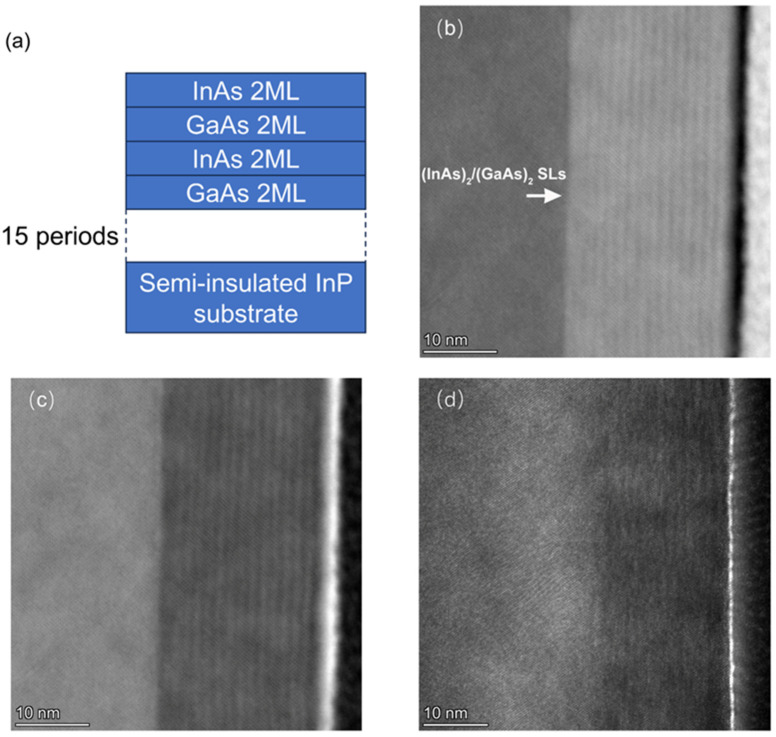
(**a**) Schematic of (InAs)_2_/(GaAs)_2_ superlattice structure with a period of 15; (**b**) HAADF-STEM image of the superlattice depicted in (**a**); (**c**) bright-field scanning transmission electron microscopy (BF-STEM) image of the superlattice illustrated in (**a**); (**d**) dark-field STEM image of the superlattice illustrated in (**a**).

The samples utilized in this study were grown by employing a Veeco Gen20A MBE system with the MEE growth mode at a low temperature of 200 °C. The GaAs or InAs layer was grown by alternately switching Ga (or In) and As shutters in a periodic manner. All samples were epitaxially grown on semi-insulating InP (100) substrates. Figure 1a illustrates the epitaxial layer for the samples investigated in this study. It is composed of 15 periods of InAs and GaAs, each consisting of two monolayers (ML) of InAs and GaAs. The crystalline structure and quality of the superlattice were characterized using a high-resolution X-ray diffractometer (HRXRD, PANanalytical, Malvern, UK, X’Pert3 MRD XL, Cu Kα1, λ = 1.54056 Å) in this study. The structure analysis of the prepared superlattice was further carried out by using the high-angle annular dark-field scanning transmission electron microscopy (Thermofisher Talos F200X, Waltham, MA, USA, HAADF-STEM) technique. The HAADF-STEM approach was used to accurately characterize the arrangement and configuration of the superlattice. The surface morphology of the grown samples was analyzed by using an atomic force microscope (AFM) (Bruker, Billerica, MA, USA, Dimension ICON, tapping mode) in this study. The electrical characteristics of the samples, including mobility and resistivity, were investigated through Hall effect measurements (Nanometrics Hall Measurement, Kanata, ON, Canada, HL5500). The carrier lifetime at room temperature was determined by the pump–probe transient reflectivity (Δ*R*/*R*) measurements. By employing an optical parametric amplifier (OPA, Opera Solo, Coherent Inc., Sunnyvale, CA, USA) that was pumped by a Ti:sapphire regenerate amplifier (Legend Elite, Coherent Inc.), the ultrashort pulse out of the OPA with the pulse width of ~150 fs and repetition rate of 1 KHz was used for the Δ*R*/*R* measurements. Both the pump and probe beams are linearly polarized, with the wavelength tuned to be nearly resonant with the bandgap of the prepared superlattice around 1.45 μm. The sample preparation for electrical characterization has been reported previously in the literature [16]. Electrical contacts were established on the sample surface as well as on the reverse side of the semi-insulating InP substrate. The measurements were performed over a temperature range of 40–290 K. Current–voltage (I-V) measurements were conducted utilizing a Keithley 4200A-SCS parameter analyzer (Keithley Instruments, Beaverton, OR, USA), with the applied voltage to the samples varying between −10 V and +20 V. The electric field distribution, as well as the photocurrent of the PCA based on the (InAs)_m_/(GaAs)_n_ superlattice structure, was derived by solving Maxwell’s equations, the drift–diffusion equation, and Poisson’s equation through finite element analysis. 

## 3. Results and Discussions

### 3.1. Crystalline Structure, Quality, and Morphology of (InAs)_m_/(GaAs)_n_ Superlattices 

In this work, a 15-cycle (InAs)_2_/(GaAs)_2_ short-period superlattice structure was grown on a semi-insulating InP substrate at a low temperature (200 °C) using the MEE growth mode of MBE. The superlattice structure of the sample is shown in Figure 1a. Figure 1b–d shows the HAADF, bright-field, and dark-field images of (InAs)_2_/(GaAs)_2_ superlattices, respectively. The results show a short-period superlattice with a period of 15, which agrees with the expected growth structure (Figure 1a). The (InAs)_2_/(GaAs)_2_ SLs, composed of alternating layers of InAs and GaAs, each with a thickness of 2 monolayers (ML), exhibit a total thickness of 19 nm.

The TEM results show a distinct and well-defined periodicity for the (InAs)_2_(GaAs)_2_ SLs, characterized by a superior crystal quality with no observable crystal defects. Remarkably, the interface of the superlattice exhibits a steep profile, indicating a well-defined structural arrangement. Usually, MBE grown at low temperatures yields sub-optimal crystal quality when compared to the growth performed at standard temperatures. This is particularly true for III-V materials grown below 200 °C, such as LT-GaAs and LT-InGaAs, which often exhibit a polycrystalline or amorphous crystalline structure with unavoidable crystal quality degradation. As a result, the relevant TEM investigations of low-temperature-grown InGaAs-based THz PCAs remain largely unexplored within the existing literature, owing to the degraded crystallization with low-temperature growth techniques.

**Figure 2:**
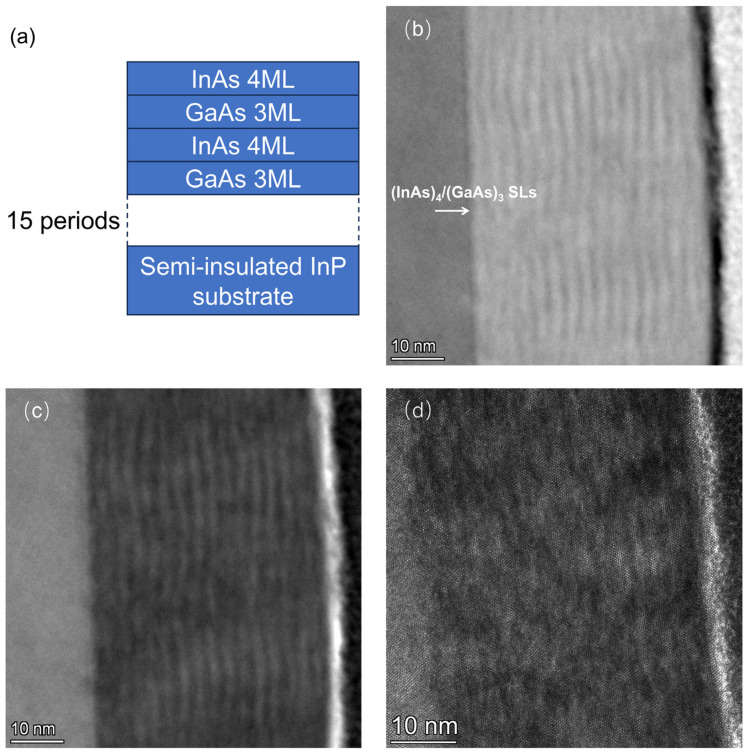
(**a**) Schematic of (InAs)_4_/(GaAs)_3_ superlattice structure with a period of 15; (**b**) HAADF-STEM image of the superlattice depicted in (**a**); (**c**) BF-STEM image of the superlattice illustrated in (**a**); (**d**) Dark-field STEM image of the superlattice illustrated in (**a**).

In this study, a 15-cycle (InAs)_4_/(GaAs)_3_ short-period superlattice structure was grown on a semi-insulating InP substrate at a low temperature of 200 °C using the MEE growth mode. The superlattice structure of the sample is shown in Figure 2a. The HAADF, bright-field, and dark-field images of the (InAs)_4_/(GaAs)_3_ superlattice are shown in Figure 2b, Figure 2c and Figure 2d, respectively. The TEM results reveal a well-defined periodicity of the (InAs)_4_(GaAs)_3_ superlattice, steep interfaces of the superlattice, and a good crystalline quality with no observable crystalline defects.

**Figure 3:**
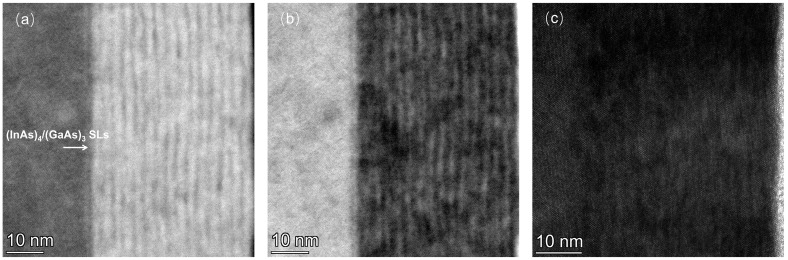
TEM image of the (InAs)_4_/(GaAs)_3_ superlattice structure after annealing. (**a**) HAADF-STEM; (**b**) BF-STEM; (**c**) dark-field STEM.

Subsequently, the (InAs)_4_/(GaAs)_3_ short-cycle superlattice samples, which were grown at a low temperature using the MEE mode, were annealed at 580 °C for 10 min. The HAADF, bright-field, and dark-field images of the annealed (InAs)_4_/(GaAs)_3_ superlattice are presented in Figure 3a–c. The TEM results indicate that the (InAs)_4_/(GaAs)_3_ superlattice maintains a well-defined periodicity after annealing, with no apparent degradation of crystal quality and no observable crystal defects.

**Figure 4:**
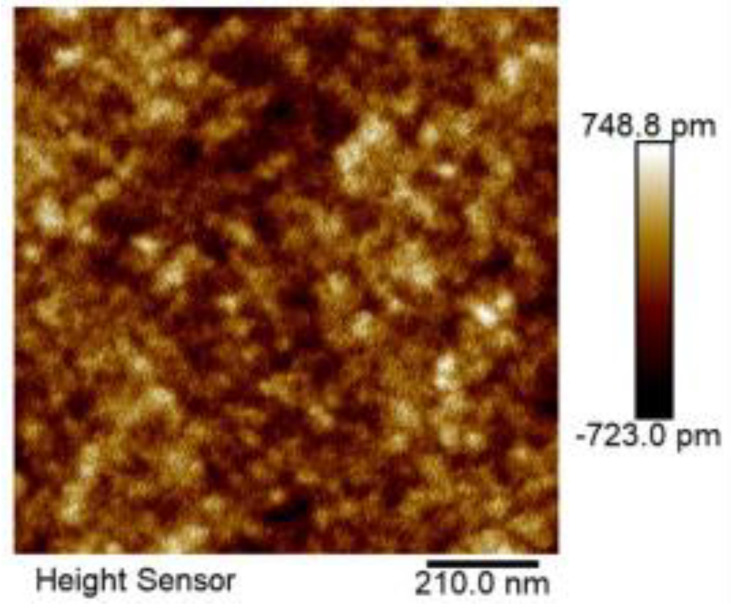
2D AFM image of a (InAs)_2_/(GaAs)_2_ superlattice sample with a scanning range of 1 × 1 μm.

**Figure 5:**
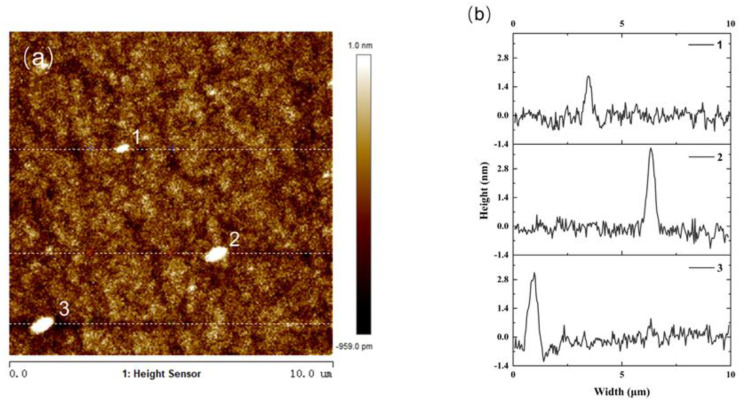
(**a**) 2D AFM image of a (InAs)_2_/(GaAs)_2_ superlattice sample with a scanning range of 10 × 10 μm; (**b**) cross-sectional profiles at points 1, 2, and 3 marked in (**a**).

Figure 4 illustrates a 2D AFM image of the superlattice sample with a scanning range of 1 × 1 μm by using tapping mode imaging. The image shows a remarkably smooth surface of the epitaxial film, characterized by a low root-mean-square roughness (*R*_q_) value of 0.167 nm. Figure 5a shows a representative 2D AFM image of a superlattice sample with a larger scanning range of 10 × 10 μm obtained also under tapping mode. The *R*_q_ value of this image is slightly elevated to 0.299 nm, and the surface roughness is slightly increased compared to the case of the same sample with a 1 × 1 μm scanning range in Figure 4, which is due to the presence of bumps 1, 2, and 3, marked in Figure 5a. Figure 5b displays the cross-sectional profiles along the scan lines corresponding to the three specific bumps (1, 2, and 3). Notably, these bumps exhibit a maximum horizontal distance of approximately 600 nm and a maximum height of around 3.5 nm. In a previous work [17], it was shown that LT-InGaAs samples grown on InP(100) substrates with a Ⅴ/III growth ratio of 29 exhibited a substantially higher *R*_q_ value of 3.9 nm by employing an AFM examination with a scanning range of 10 × 10 μm. Additionally, the surface of these samples displayed pronounced roughness, characterized by the presence of numerous pits. By comparing these results with our studies, it is evident that the superlattice samples grown in this study exhibit a superior surface quality with a much-improved smoothness.

The AFM image presented in this work illustrates the surface morphology of an (InAs)_2_/(GaAs)_2_ multi-period superlattice grown on an InP substrate. The growth mode employed involved the alternate switching on and off of the shutters of Group In(Ga) and Group As, thereby enhancing the migration of atoms at low temperatures and facilitating the growth of the (InAs)_2_/(GaAs)_2_ multi-period superlattice at 200 °C. However, it is important to note that the adatom’s migration velocity is also affected by the decrease in temperature. We attribute the observed bumps in the AFM image to the localized stress relaxation at individual points during the low-temperature growth process. Specifically, at reduced temperatures, the limited mobility of the adatoms restricted their ability to reach equilibrium positions on the growing surface, leading to inhomogeneous nucleation sites. As the growth progressed, these nucleation sites evolved into three-dimensional nanostructures, which manifest as the bumps observed in the AFM image.

**Figure 6:**
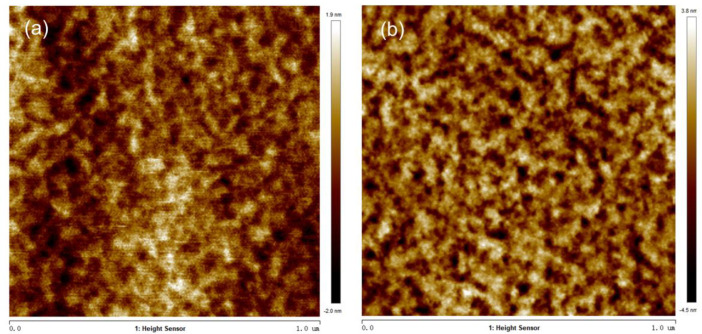
AFM images of (InAs)_4_/(GaAs)_3_ superlattice samples: (**a**) as-grown; and (**b**) after annealing at 580 °C.

Figure 6 shows the AFM images of the (InAs)_4_/(GaAs)_3_ samples before and after annealing, with the *R*_q_ value of 0.565 nm for the as-grown samples, and 1.23 nm after annealing at 580 °C. The increased surface roughness of samples annealed at high temperatures is mainly attributed to surface desorption and atomic migration.

### 3.2. HRXRD of (InAs)_m_/(GaAs)_n_ Superlattice Structures 

**Figure 7:**
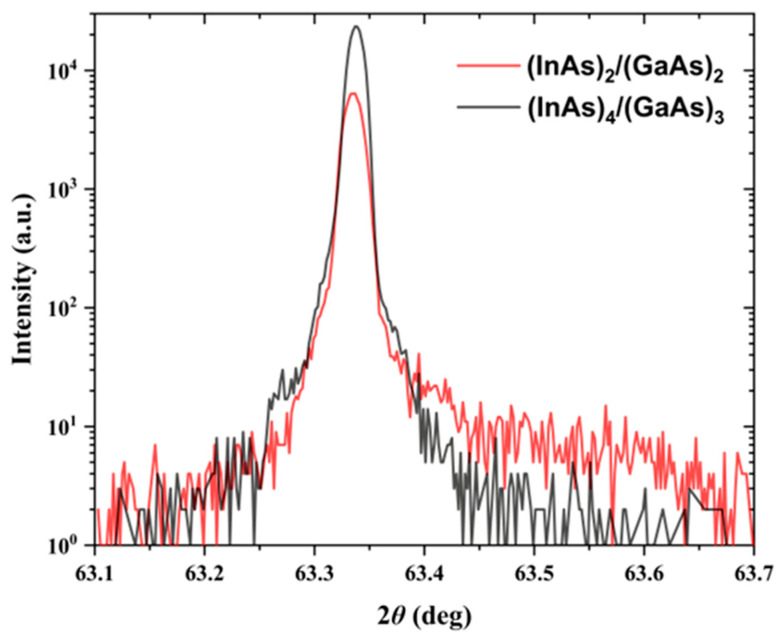
HRXRD patterns of (InAs)_2_/(GaAs)_2_ and (InAs)_4_/(GaAs)_3_ superlattice samples obtained in the *θ*/2*θ* scanning mode.

As shown in Figure 7, the 2*θ* diffraction peak of the InP substrate in the (InAs)_4_/(GaAs)_3_ and (InAs)_2_/(GaAs)_2_ superlattice samples is at 63.337°, which is consistent with the standard diffraction peak position of the InP (004) crystal plane, as seen in the HRXRD curves [17]. The XRD pattern in Figure 7 presents the original, unnormalized test results, showing some differences in the intensity values of the InP (004) crystal plane peak between the (InAs)_4_/(GaAs)_3_ and (InAs)_2_/(GaAs)_2_ short-period superlattices.

The (InAs)_4_/(GaAs)_3_ short-period superlattice has an equivalent In composition of 0.537, closely matching the InP (001) substrate, resulting in a diffraction peak that falls within the envelope of the InP (004) plane’s peak at 63.337°, making it indistinguishable in the graph. No additional diffraction peaks were observed for the (InAs)_4_/(GaAs)_3_ superlattice samples, indicating that the (InAs)_4_/(GaAs)_3_ superlattice is lattice-matched to the substrate.

The broad peaks between 63.4° and 63.7° are attributed to the (InAs)_2_/(GaAs)_2_ short-period superlattice layer, with a thickness of approximately 19 nm. The equivalent In composition of the (InAs)_2_/(GaAs)_2_ superlattice is 0.465, corresponding to the XRD peaks within the mentioned broad peak range. The lower intensity of these peaks is attributed to the thinness of the film, resulting in a weaker XRD signal. According to Scherrer’s formula, thinner films correspond to broader peaks. The observed broadening of the diffraction peak in Figure 7 for the (InAs)_2_/(GaAs)_2_ superlattice is ascribed to the relatively thin film thickness, based on the following considerations. In our analysis, we have utilized the Scherrer formula, which is commonly employed to estimate the crystallite size and understand the broadening of X-ray diffraction (XRD) peaks in thin film materials. According to the Scherrer formula, when two-dimensional film materials are relatively thin, the XRD peaks are expected to exhibit broadening due to the finite size of the crystallites in the direction perpendicular to the film surface. For the (InAs)_2_/(GaAs)_2_ superlattice presented in Figure 7, we have calculated the full width at half-maximum (FWHM) of the XRD peak using the Scherrer formula. Our calculations yield a FWHM of 0.4°, which is in agreement with the peak width observed in the XRD pattern. Furthermore, the TEM analysis presented in Figure 1 confirms the high crystalline quality of the (InAs)_2_/(GaAs)_2_ superlattice. This analysis reveals the absence of significant crystal dislocations and defects within the superlattice, indicating a high level of structural integrity and crystalline quality.

Therefore, the broad diffraction peak observed in the HRXRD curve, as shown in Figure 7 for the (InAs)_2_/(GaAs)_2_ superlattice, is attributed to its relatively thin thickness, rather than the crystal dislocations and defects. 

**Figure 8:**
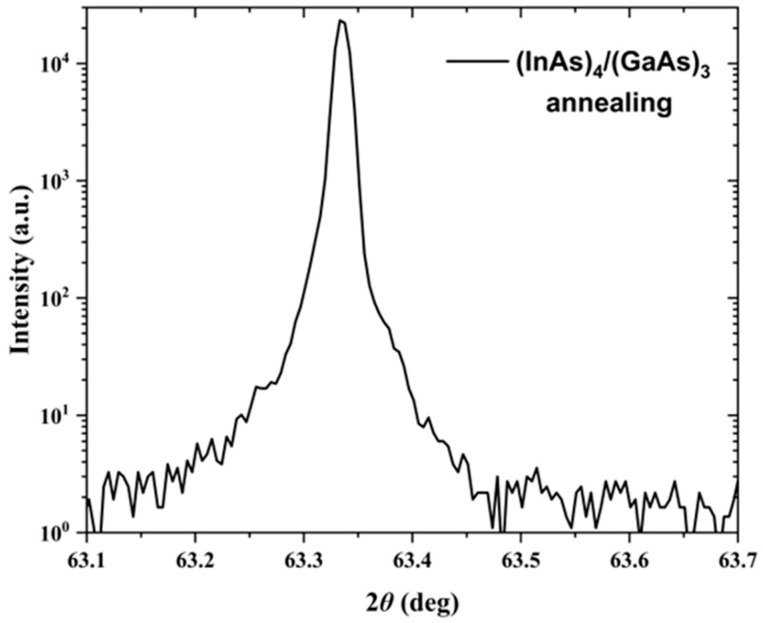
HRXRD patterns of (InAs)_4_/(GaAs)_3_ superlattice samples after annealing obtained in the *θ*/2*θ* scanning mode.

The HRXRD results of the annealed (InAs)_4_/(GaAs)_3_ superlattice samples, shown in Figure 8, exhibit peak shapes similar to those observed in the HRXRD curves of the as-grown sample, with no significant broadening evident in the diffraction peaks. This indicates that the crystal quality of the annealed (InAs)_4_/(GaAs)_3_ superlattice samples remains relatively high, with no apparent degradation.

Based on the HRXRD results, it is evident that the (InAs)_4_/(GaAs)_3_ superlattice samples are lattice-matched to the InP (100) substrate and are suitable for growing thicker layers. This makes them suitable for the fabrication of PCA antennas. Therefore, the rest of the paper will focus on this class of superlattice samples.

### 3.3. Raman Spectra of (InAs)_m_/(GaAs)_n_ Superlattice Structures 

**Figure 9:**
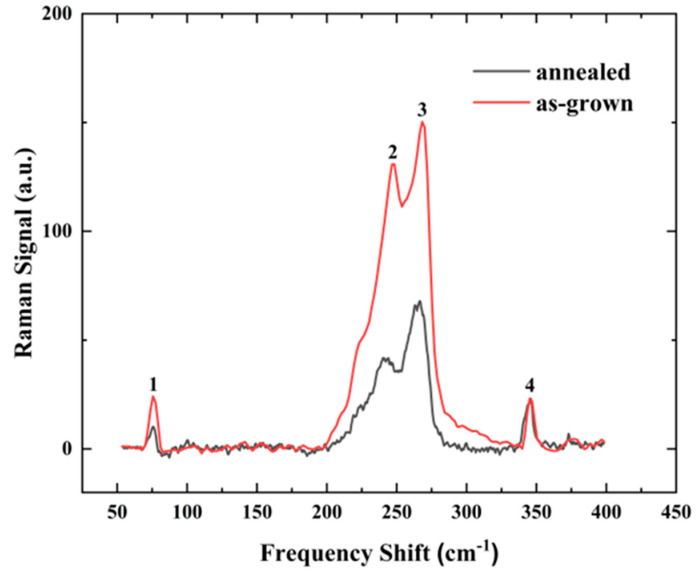
Raman spectra of (InAs)_4_(GaAs)_3_ superlattice samples before and after annealing.

It is known that the Raman spectrum can reveal the characteristic phonon information and interface perfection of the superlattice [18]. The longitudinal acoustic (LA) modes can be observed in the center of the Brillouin zone, providing information about the periodicity of the superlattice. The localization of the longitudinal optical (LO) phonons at the superlattice interfaces can help researchers study the composition of the individual layers [19]. To further characterize the fabricated (InAs)_4_/(GaAs)_3_ superlattice grown in this study, the Raman spectra were acquired for both the as-grown and annealed samples using a 532 nm laser excitation with a scanning range from 50 to 400 cm^−1^, as shown in Figure 9. The Raman peaks in Figure 9 indicate the presence of superlattices. The Raman peaks at 345 cm^−1^ (labeled as 4) and 269 cm^−1^ (labeled as 3) are attributed to the LO mode of the InP substrate and the transverse optical (TO) phonon mode of GaAs, respectively. Another Raman peak labeled as 2 is attributed to a propagating optical mode primarily associated with InAs [20]. These peaks are affected by the strain (GaAs and InAs strain-matched InP) and quantum confinement in the (InAs)_4_(GaAs)_3_ short superlattice. In addition, there is a low-energy Raman peak at 75 cm^−1^ attributed to the LA mode, which is related to the periodicity of the superlattice. This peak’s position is close to that of the LA mode of a (InAs)_4_(GaAs)_3_ superlattice measured at 77 K, as reported in the literature [14]. This implies that we obtained the same periodicity of the superlattice as that in [14]. This is because the LA mode, corresponding to the lowest frequency mode of the lattice vibrations at the center of the Brillouin zone, can be used to evaluate the periodic structure of the superlattice. Moreover, the peak positions of the LA modes are mostly stable over a typical temperature range without significant temperature dependence, since the LA modes are mainly affected by the elastic properties of the lattice and its geometrical configuration, with relatively minor temperature variation. Nevertheless, our TEM and Raman results indicate the sharp interfaces and excellent crystal quality for the fabricated superlattices. The individual peak positions of the annealed sample are basically the same compared to those of the as-grown sample, and there is no obvious change in the FWHM, indicating that the crystal quality of the (InAs)_4_/(GaAs)_3_ superlattice does not change significantly after annealing, and there still exists an obvious periodicity with an unchanged period, which is consistent with the TEM results after annealing (Figure 3). The weakening of the 1, 2, and 3 peak signals after annealing may be related to the decrease in Raman signal collection efficiency due to the increase in surface roughness or the slight degradation of crystal quality with high-temperature annealing.

### 3.4. Hall Effect and Carrier Lifetime Measurements of (InAs)_m_/(GaAs)_n_ Superlattices

The resistivity, mobility, and carrier lifetime, as the key material parameters for making a superior photoconductive antenna, depend greatly on the crystal quality of the epitaxial structure. To evaluate the electrical properties of the samples, a 15-cycle (InAs)_4_/(GaAs)_3_ superlattice structure was annealed at 580 °C in an H_2_-protecting environment for 10 min. Hall measurements at room temperature showed that the as-grown sample had a mobility of 843 cm^2^/(V·s) and a square resistance of 1648 ohm/sq, while the annealed sample exhibited a reduced mobility of 766 cm^2^/(V·s) and a significant increase in square resistance to 53,887 ohm/sq. The room temperature mobility was reported to be only 425 cm^2^/(V·s) for the Be-doped LT-InGaAs annealed at 580 °C [21]. The enhanced mobility observed in this study can be attributed to the utilization of (InAs)_4_/(GaAs)_3_ short-period superlattices instead of InGaAs, so that the lattice scattering resulting from the inherent disorder in the ternary alloy can be effectively reduced. The increase in the squared resistance is attributed to the formation of As clusters precipitated during the high-temperature annealing process, resulting in a Schottky barrier around the As precipitate, which leads to an elevation in the resistivity.

**Figure 10:**
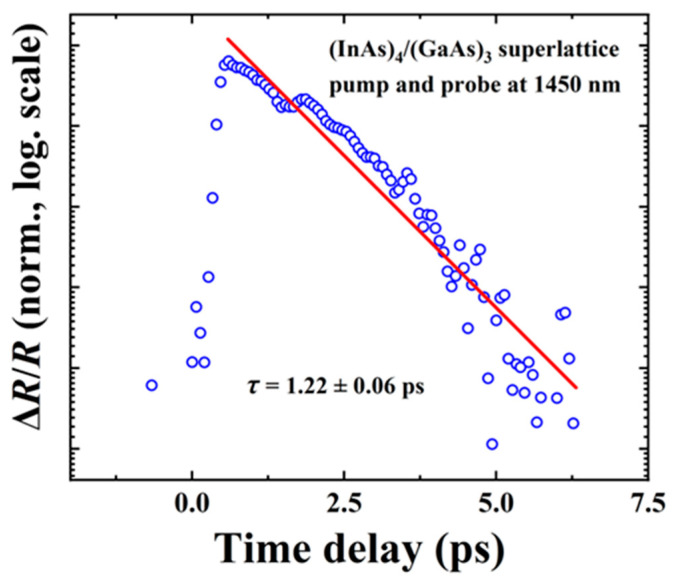
The pump–probe transient reflectivity result for (InAs)_4_/(GaAs)_3_ superlattice sample at room temperature. The measurement is performed at excitation wavelength of 1450 nm. The scattered blue circles are the experimental data and the red solid line is fitting results by using a single exponential decay function.

The typical pump–probe transient reflectivity result, measured under an excitation wavelength of 1450 nm with a pumping power of 6 mW at room temperature, is shown in Figure 10. The decay curve represents the carrier lifetime (*τ*) of the superlattice, providing insights into the recombination dynamics of carriers within the structure. The carrier lifetime of the (InAs)_4_(GaAs)_3_ superlattice samples was determined to be approximately 1.2 ps by fitting with a single exponential decay function. In the study conducted by Namje Kim et al. [15], the carrier lifetime of a Be-doped LTG-InGaAs sample grown on a semi-insulating InP substrate at 250 °C was measured to be 7.8 ps. It is clear that the (InAs)_m_/(GaAs)_n_ superlattice structure prepared in this work exhibits a significantly shorter carrier lifetime.

**Figure 11:**
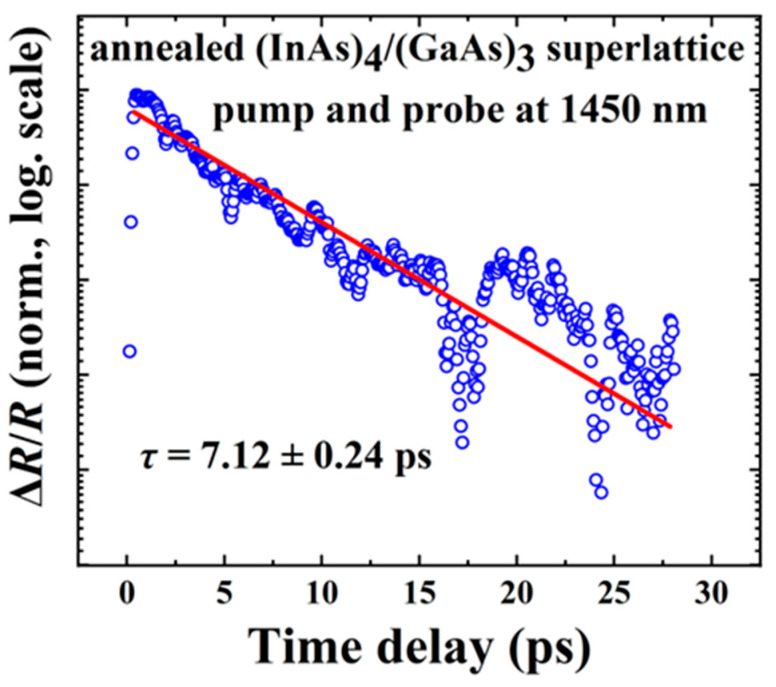
The pump–probe transient reflectivity result at room temperature for (InAs)_4_/(GaAs)_3_ superlattice sample after annealing. The measurement is performed at excitation wavelength of 1450 nm. The scattered blue circles are the experimental data and the red solid line is fitting results by using a single exponential decay function.

The typical pump–probe transient reflectivity result for the (InAs)_4_/(GaAs)_3_ superlattice sample after annealing is shown in Figure 11, measured under an excitation wavelength of 1450 nm with a pumping power of 6 mW at room temperature. The carrier lifetime of the (InAs)_4_/(GaAs)_3_ superlattice sample was determined to be approximately 7.1 ps by fitting with a single exponential decay function. The increased carrier lifetime in the (InAs)_4_(GaAs)_3_ superlattice samples after annealing is attributed to the high-temperature annealing process, which helps remove defects in the crystal such as As_Ga_ antisite defects that act as non-radiative recombination centers. During the annealing process, the migration and elimination of crystal defects is facilitated, leading to a reduction in these non-radiative recombination centers and thus to a longer carrier lifetime.

### 3.5. Electrical Properties of (InAs)_m_/(GaAs)_n_ Superlattices at Different Temperatures

**Figure 12:**
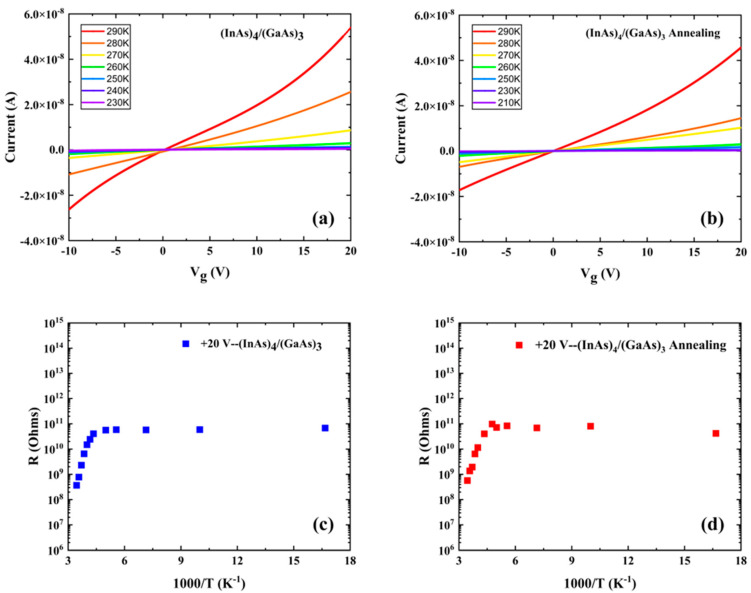
Electrical properties of (InAs)_4_/(GaAs)_3_ superlattices at different temperatures. (**a**) I-V characteristics of as-grown sample; (**b**) I-V characteristics of annealed sample; (**c**) resistance of as-grown sample; and (**d**) resistance of annealed sample.

The electrical properties of the as-grown and annealed (InAs)_4_/(GaAs)_3_ superlattices at different temperatures are shown in Figure 12. Figure 12a,b represents the current–voltage (I-V) characteristics of the vertical current transport through the as-grown and annealed (InAs)_4_/(GaAs)_3_ superlattices, respectively, as a function of the applied voltage under different temperatures. The I-V curves were obtained by applying a voltage across the top surface of the superlattice sample and the bottom of the InP substrate at various temperatures. The results in the figure show that the current through both the as-grown and annealed samples decreases as the temperature decreases. The current saturates at 230 K for the as-grown sample, while it saturates at a slightly lower 210 K for the annealed sample. The elevated current observed in the as-grown sample, as compared to the annealed sample at equivalent voltages, can be attributed to the increased resistance in the latter.

Figure 12c,d presents the temperature-dependent resistance behaviors for the as-grown and annealed superlattice samples, respectively, under an applied sample surface gate voltage (Vg) of +20 V. For the applied electric field (Vg ± 20 V), the resistivity curves have one distinct slope in the high-temperature region, and the activation energies corresponding to this slope are 294 meV and 332 meV for as-grown and annealed samples, respectively. This value closely approximates the position of the electron level in the conduction band of the (InAs)_4_/(GaAs)_3_ superlattice (about 0.28 eV). No significant slope is found in the low-temperature region, and the situation is similar in the range of applied voltages between −10 V and +20 V. The thermal activation energies in the high-temperature region of the as-grown and annealed samples are close to each other, and the thermal activation energy of the annealed sample is slightly higher, probably due to the formation of clusters from excess As in the low-temperature-grown (InAs)_4_/(GaAs)_3_ superlattice after annealing and an increase in resistance. Aggregated arsenic atoms may introduce localized states that impede charge transport, necessitating additional thermal energy to excite charge carriers across the enhanced activation gap and hence increase resistance.

Tunneling effects in nanoscale multilayer electrical transport were investigated by V Osinniy et al. [16]. In their study, three distinct tunneling mechanisms were discussed: photon-assisted tunneling, direct tunneling, and Poole–Frenkel tunneling. In this paper, voltages were applied across the samples consisting of an (InAs)_4_/(GaAs)_3_ superlattice and a semi-insulating InP substrate. Compared to the electric field applied to the samples in reference [16], the electric field imposed on the (InAs)_4_/(GaAs)_3_ superlattices here was lower. Therefore, the vertical current measurements were performed at relatively low electric field conditions. As reported in the literature [16], it is suggested that phonon-assisted and direct tunneling mechanisms are significant contributors to the vertical transport of charge carriers under the conditions of low electric fields.

### 3.6. Simulation of Terahertz PCA 

#### 3.6.1. Theory

The THz intensity radiated from a THz PCA is correlated with the transient optical current density *J_s_*(*t*) generated in the PCA by laser excitation. Under far-field conditions, the THz electric field can be written as the following [22]:

(1) $$E_{THz}\left(z,t\right)=-\frac{\mu_{0}A}{4\pi z}\frac{dJ_{s}\left(t\right)}{dt}$$

here, *μ*_0_, *A*, *z*, and *J_s_*(*t*) represent the permeability of free space, the area illuminated by the laser between the antenna electrodes, the distance from the center of THz PCA radiation to the measurement point of THz electric field, and the current density on the surface of the THz PCA, respectively. From the aforementioned equation, the electric field intensity of terahertz radiation is directly proportional to the temporal derivative of the surface current of the PCA. In this study, the Maxwell wave equation and the carrier kinetic equation are solved using the finite element method. This approach allows for a more precise investigation of the effect of the electrode structure on the photoelectric response of the PCA.

#### 3.6.2. Simulation of Terahertz PCA Based on (InAs)_m_/(GaAs)_n_ Superlattice Structure

The optical response of a PCA is obtained by solving the frequency-domain equations for electromagnetic waves [23]:

(2) $$\nabla \times \mu_{r}^{-1}\left(\nabla \times E\right)-k_{0}^{2}\left(\varepsilon_{r}-\frac{j\lambda \sigma }{2\pi c\varepsilon_{0}}\right)E=0$$

where *ε*_0_, $\varepsilon_{r}$, *σ*, $u_{r}$, $k_{0}$, *λ*, *c*, and **E** represent the permittivity of free space, relative permittivity, electrical conductivity, relative magnetic permeability, free-space propagation constant, incident laser wavelength, light velocity in the vacuum, and complex electric field vector of the PCA, respectively. The spatial distribution of the laser beam follows a Gaussian profile, and the temporal evolution of the photogenerated carriers within the PCA after excitation with a Gaussian pulse can be described as the following [23,24]:

(3) $$G\left(x,y,z,t\right)=\frac{4\pi k_{pc}}{hc}P_{s}\left(x,y,z\right)exp\left(4ln\left(0.5\right)\cdot \frac{{\left(t-t_{0}\right)}^{2}}{D_{t}^{2}}\right)$$

here $k_{pc}$, h, $D_{t}$, *t*_0_, and *P_s_*(*x*, *y*, *z*) represent the imaginary part of the refractive index of the PCA substrate material, Planck’s constant, the duration of the laser pulse (pulse width), the central time of the pulse, and the total power flux density of the PCA, respectively. The photo-generated carrier density was obtained by analyzing the optical response and the transient photocurrent was determined by solving the time-dependent carrier kinetic equation.

**Figure 13:**
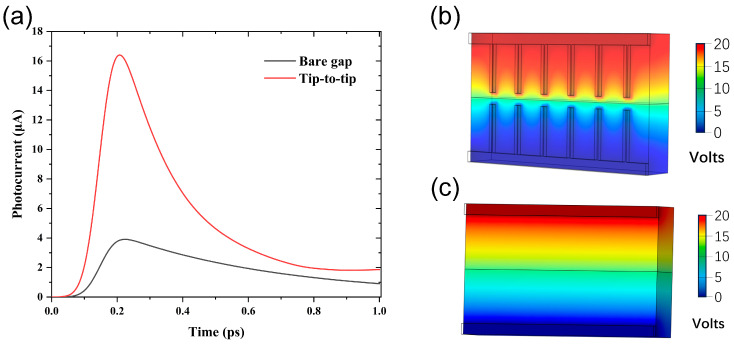
Simulated transient photocurrent response and potential distribution images of bare gap and tip-to-tip PCAs under laser irradiation at 800 nm, with a laser intensity of 10 mW, pulse width of 60 fs, and a repetition rate of 80 MHz; (**a**) transient photocurrent of bare gap and tip-to-tip PCAs; (**b**) potential distribution of tip-to-tip PCA; and (**c**) potential distribution of bare gap PCA.

To examine the accuracy of the models employed in this study, we first conducted the transient photocurrent and potential distribution evaluation by the model employed in this study for both types of PCA, namely the bare gap and tip-to-tip configurations. To ensure a fair comparison, our simulations were performed using the identical material, shape, and size parameters as reported by Emadi and Safian et al. [25]. According to their findings, the maximum transient photocurrent values obtained for the bare gap antenna and tip-to-tip electrode antenna were approximately 4 × 10^−6^ A and 17 × 10^−6^ A, respectively. These results were obtained with a laser intensity of 10 mW and a bias voltage of 20 V. As presented in Figure 13, our simulation results obtained in this study are close to those reported in the literature [25], implying the validity and accuracy of the model employed in this study.

**Figure 14:**
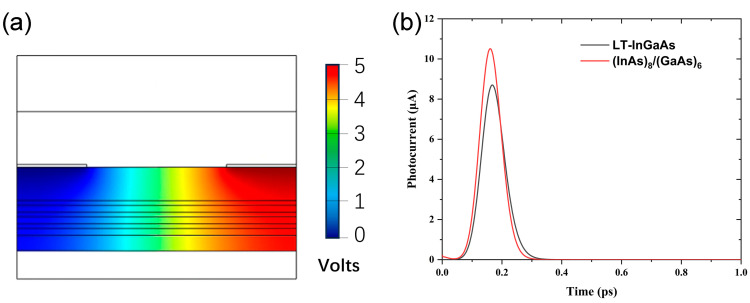
(**a**) Potential distribution image of a single quantum well structure consisting of a 3-cycle (InAs)_8_/(GaAs)_6_ superlattice as a quantum well and a 12 nm wide In_0.52_Al_0.48_As as a barrier; and (**b**) transient photocurrent in (InAs)_8_/(GaAs)_6_ and LT-InGaAs/InAlAs single quantum well structures.

After the successful growth of good-quality superlattice structures as addressed above, we further fabricated the quantum well structure by incorporating the superlattice structure presented above. The single quantum well structure comprises a 3-cycle (InAs)_8_/(GaAs)_6_ superlattice serving as the quantum well, with a 12 nm wide In_0.52_Al_0.48_As layer acting as the barrier. Its potential distribution and transient photocurrent response are then simulated with irradiation of a femtosecond laser at 1550 nm, as shown in Figure 14. The other parameters used in our simulation can be found in Table 1. In Figure 14b, the transient photocurrent evaluated by using the aforementioned model was compared between the (InAs)_8_/(GaAs)_6_ and LT-InGaAs/InAlAs quantum well structures. Here, the quantum well width of the LT-InGaAs/InAlAs structure is equivalent to that of the 3-cycle (InAs)_8_/(GaAs)_6_ structure, with the same In component as well. It can be seen in Figure 14b that the transient photocurrents of the (InAs)_8_/(GaAs)_6_ single quantum well structure are approximately 10.51 × 10^−6^ A, while those of the LT-InGaAs single quantum well are approximately 8.70 × 10^−6^ A. The slightly higher transient photocurrent in the (InAs)_8_/(GaAs)_6_ single quantum well can be attributed to the greatly suppressed alloy scattering in the (InAs)_8_/(GaAs)_6_ single quantum well compared to that of the LT-InGaAs; therefore, a higher mobility and transient photocurrent are expected in the (InAs)_8_/(GaAs)_6_ single quantum well structure.

Comparing the results in Figure 14 with those presented in Figure 13, it is obvious that the simulated transient photocurrents for both the (InAs)_8_/(GaAs)_6_ and LT-InGaAs quantum well materials are relatively low. This is because our simulation is done only for a single narrow quantum well structure, limited by the required high computational capacity for complex quantum well structures. Nevertheless, our simulation results indicate that even the single-period of the (InAs)_8_/(GaAs)_6_ quantum well can generate significant transient photocurrents. In our future investigations, we will continue to optimize the low-temperature growth of the (InAs)_m_/(GaAs)_m_/InAlAs multiple quantum wells using the MEE mode of MBE, and further enhance the THz radiation power.

## 4. Conclusions

In conclusion, this study demonstrates the successful growth of high-quality (InAs)_m_/(GaAs)_n_ short-period superlattices by employing the MEE mode of molecular beam epitaxy. The electron mobility and resistivity at room temperature for the prepared (InAs)_m_/(GaAs)_m_ superlattices are determined to be 843 cm^2^/(V·s) and 1648 ohm/sq, respectively. Additionally, the band-edge photo-excited carrier lifetime is approximately 1.8 ps at room temperature. The calculated photocurrent intensity of a (InAs)_8_/(GaAs)_6_ single quantum well structure can reach up to approximately 10.46 × 10^−6^ A. The improved electrical properties and higher photocurrent generation in (InAs)_m_/(GaAs)_m_ superlattices compared to that of LT-InGaAs suggests that the MEE epitaxial growth technique can be a promising approach to realizing high-performance THz PCAs with enhanced radiation power. 

## Figures and Tables

**Table 1:** Physical parameters of the simulated LT-GaAs and InAs.

Parameter	Value
Operating temperature	300 K
Bandgap of LT-GaAs	1.424 V
Bandgap of InAs	0.354 V
Electron affinity of LT-GaAs	4.07 V
Electron affinity of InAs	4.9 V
Low-field electron mobility of LT-GaAs	4 × 10^2^ cm^2^/(V·s)
Low-field hole mobility of LT-GaAs	1 × 10^2^ cm^2^/(V·s)
Low-field electron mobility of InAs	4 × 10^4^ cm^2^/(V·s)
Low-field hole mobility of InAs	5 × 10^2^ cm^2^/(V·s)
Carrier lifetime	1.77 ps
Effective conduction band density of states of LT-GaAs	4.7 × 10^17^ 1/cm^3^
Effective valence band density of states of LT-GaAs	9.0 × 10^18^ 1/cm^3^
Effective conduction band density of states of InAs	6.6 × 10^18^ 1/cm^3^
Effective valence band density of states of InAs	8.7 × 10^16^ 1/cm^3^
Profile of incident light	Gaussian

## Data Availability

The data supporting the findings of this study are available from the corresponding author upon reasonable request.
